# Medical comorbidities in Saudi patients with narcolepsy: a case-control study

**DOI:** 10.5935/1984-0063.20200070

**Published:** 2021

**Authors:** Ahmed Salem BaHammam, Awad H Olaish, Majid Al-Omar, Aljohara S Almeneessier

**Affiliations:** 1 King Saud University, University Sleep Disorders Center, Department of Medicine, College of Medicine - Riyadh - Riyadh - Saudi Arabia.; 2 Strategic Technologies Program of the National Plan for Sciences and Technology and Innovation in the Kingdom of Saudi Arabia, National Plan - Riyadh - Riyadh - Saudi Arabia.; 3 King Saud University, Family and Community Medicine Department - Riyadh - Riyadh - Saudi Arabia.

**Keywords:** Hypertension, Sleep, Diabetes Mellitus, Disorders of Excessive Somnolence, Hyperlipidemias, Hypothyroidism

## Abstract

**Objective:**

This case-control study sought to assess comorbid medical disorders in patients with narcolepsy type-1 (NT-1) and type-2 (NT-2).

**Material and Methods:**

The study comprised 80 consecutive Arab (Saudi) patients with narcolepsy (NT-1=56 and NT-2=24) and a control group of 211 adults matched for age, sex, and body mass index (BMI). Data were collected from cases and controls based on a predesigned questionnaire that was formulated based on previous studies to evaluate the chosen medical comorbidities.

**Results:**

Narcolepsy patients had a higher prevalence of hypothyroidism and hyperlipidemia and a higher prevalence of high-risk for OSA than controls. Hyperlipidemia was more common in cases than controls, 8 (10%) vs. 3 (1.4%), p=0.002. After adjusting for age, sex, and BMI, the odds-ratios for hypothyroidism and high risk for OSA in the NT-1 group was 5.49 (95% CI, [0.8 - 38.6]) and 69.99 ((95%CI [20.6 -237.4]), respectively, and in the NT-2 group, 12.5, 95%CI [1.6-97.7], and 33.3, 95%CI [8.2-135.7], respectively.

**Conclusion:**

Arab (Saudi) narcolepsy patients had a higher association with hypothyroidism, hyperlipidemia, and a higher risk of OSA than controls.

## INTRODUCTION

Narcolepsy is a chronic sleep disorder that ensues secondary to the degeneration of orexin (hypocretin), producing neurocytes in the lateral hypothalamus^1^. Currently, narcolepsy diagnosis follows the criteria of the International Classification of Sleep Disorders, third edition (ICSD-3), where all patients have irresistible attacks of sleep, and they may have other symptoms like cataplexy, hypnagogic or hypnopompic hallucinations, sleep paralysis, and sleep-maintenance insomnia^2^. The ICSD-3 divides narcolepsy into narcolepsy type-1 (NT-1) (with cataplexy) and narcolepsy type-2 (NT-2) (without cataplexy). The estimated prevalence of narcolepsy in Saudi Arabia is approximately 0.04%^3^.

A few studies have reported that narcolepsy may have a higher association with medical comorbidities than matched controls, such as thyroid disease, sleep apnea, hyperlipidemia, hypertension, and diabetes^4-9^. We have previously reported the comorbid psychiatric and autoimmune diseases in Arabs (Saudis) with narcolepsy^1,10^, but no study has reported the prevalence of comorbid medical comorbidities in Saudis or Arabs.

We hypothesized that comorbid medical disorders are higher in Arab (Saudi) patients with narcolepsy compared to matched controls. Therefore, this case-control study sought to assess comorbid medical disorders in patients with narcolepsy (NT-1 and NT-2).

## MATERIAL AND METHODS

In this case-control study, narcolepsy patients and controls, matched for age, sex, and body mass index (BMI) were included. Eighty consecutive narcolepsy patients who visited the sleep disorders clinic between 1 September 2016 and 31 December 2017 were included. Controls (n=211) were recruited during the same period from public places.

The diagnosis of narcolepsy followed the ICSD-3 diagnostic criteria^2^. A standard type-1, in laboratory attended overnight polysomnography (PSG) was performed for all patients, followed by a standard multiple sleep latency test (MSLT) to assess sleep latency and sleep-onset rapid eye movement periods (SOREMs)^2^. Among narcolepsy patients who met the ICSD-3 diagnostic criteria, if clear cataplexy (“more than one episode of generally brief (<2 min), usually bilaterally symmetrical, sudden loss of muscle tone with retained consciousness”) was present, NT-1 was diagnosed. NT-2 was diagnosed when there was a mean latency of <8 min on the MSLT and two SOREMPs (or one SOREMP on PSG and one or more on MSLT), but without cataplexy^2^.

Data were collected from cases and controls based on a predesigned questionnaire that was formulated on the basis of previous studies to evaluate the chosen medical comorbidities^1,4-9^. Medical comorbid conditions were diagnosed in cases and controls based on medical history and medical reports from other specialists. Comorbidities were confirmed, when feasible, via checking the participants’ electronic medical records. Based on previous studies that reported comorbidities in narcolepsy, the search included the following medical disorders, diabetes mellitus type-2, hypertension, hyperlipidemia, hypothyroidism, obstructive sleep apnea (OSA), and epilepsy^4-9^. The risk for OSA was assessed in cases and controls using a validated Arabic version of the Berlin Questionnaire (a validated questionnaire that categorizes individuals into high-risk and low-risk for OSA)^11,12^. For patients with narcolepsy and comorbid OSA, the MSLT was done after treatment with positive airway pressure to minimize the effect of OSA on the measured sleepiness.

All participants signed a written informed consent form, and the ethics committee in the College of Medicine at King Saud University approved the study proposal.

### Statistical analysis

Data were presented as means ± standard error (SE) for continuous variables and percentages for dichotomous variables. For comparing groups, the Chi-square test or the Fisher’s exact test were used for dichotomous data and the Student’s t-test for continuous data with a normal distribution. The Mann-Whitney U test was used if normality failed. For comparing NT-1, NT-2, and controls, one-way analysis of variance (ANOVA) was used, and if the normality test failed, one-way ANOVA on ranks was used.

Cases and control were matched for age, BMI, and sex. However, upon dividing the cases into NT-1 and NT-2, there was a mismatch between the two subgroups of cases and controls in age, BMI, and sex. Therefore, a univariate logistic regression analysis was applied to evaluate cases and controls for comorbid medical conditions exposure variables and was presented as odds ratios (OR) and the 95% confidence intervals (CI). An initial “model-0” as crude associations was performed between cases and controls. Then a regression analysis, after adjusting for age, BMI, and sex was performed “model-1”.

The Statistical Package for Social Sciences software version 23.0 was used for the analysis (SPSS Inc., Chicago, Illinois, USA) and *p*-values ≤0.05 were considered significant.

## RESULTS

Table 1 presents the important polysomnographic and MSLT parameters of patients with NT-1 and NT-2. On the MSLT, sleep latency for NT-1, and NT-2 was 2.3±0.5 min and 3.3±0.6 min, respectively, and the average SOREMS for NT-1 and NT-2 were 3.7±0.2 and 3.1±0.3, respectively.

**Table 1.** Polysomnographic and multiple sleep latency rest recordings of patients with narcolepsy.

Variables	Narcolepsy type-1 (n=56)	Narcolepsy type-2 (n=24)
**Epworth sleepiness scale**	19.1 ± 3.6	16.5 ± 5.2
**Polysomnographic findings**		
Sleep latency (min)	5.6 ± 1.3	7.3 ± 2.4
Latency to rapid eye movement (min)	52.9 ± 14.1	93.1 ± 19.8
Sleep efficiency (%)	78.9 ± 6.6	85.1 ± 6.3
Arousal index	30.1 ± 7.3	17.1 ± 5.1
**Multiple sleep latency test**		
Sleep latency (min)	2.3 ± 0.5	3.3 ± 0.6
Sleep onset rapid eye movement periods (average)	3.7 ± 0.2	3.1 ± 0.3
Rapid eye movement latency (min)	2.6 ± 0.3	4.4 ± 0.8

No differences were detected between the whole group of narcolepsy patients and controls concerning age, sex, and BMI. The mean age and BMI for cases and controls were 34.6±1.6 years vs. 36.6±0.9 years, and 28.6±0.3kg/m^2^ vs. 27.7±0.4kg/m^2^, respectively. Women represented 83.8% of cases and 75% of controls (Table 2). However, when narcolepsy patients were divided into NT-1 and NT-2 (Table 2), the NT-1 group had some differences compared to controls in age (32±1.9 years vs. 36.6±0.9 years, *p*=0.001 and BMI (29.7±0.9kg/m^2^ vs. 27.7±0.4kg/m^2^, *p*=0.02). NT-1 represented 70% (n=56) and NT-2 30% (n=24) of narcolepsy patients.

**Table 2.** A comparison between cases and controls.

Variable	Total (n=291)	Mean ± SE/n (%)		P-Value
		Case (n=80)	Control (n=211)	
		
Age (Year)	36 ± 0.8	34.6 ± 1.6	36.6 ± 0.9	0.14
Age groups				
< 21	19 (6.5)	7 (8.8)	12 (5.7)	0.78
21-30	109 (37.5)	32 (40)	77 (36.5)	
31-40	80 (27.5)	19 (23.8)	61 (28.9)	
41-50	35 (12)	10 (12.5)	25 (11.8)	
> 50	48 (16.5)	12 (15)	36 (17.1)	
Sex (Females)	225 (77.3)	67 (83.8)	158 (74.9)	0.14
Body Mass Index (kg/m2)	28 ± 0.3	28.6 ± 0.7	27.7 ± 0.4	0.19
Hypothyroidism	7 (2.4)	5 (6.3)	2 (0.9)	0.02
High-risk of OSA	41 (14.1)	38 (47.5)	3 (1.4)	<0.001
Type 2 diabetes mellitus	30 (10.3)	8 (10)	22 (10.4)	0.9
Hypertension	18 (6.2)	2 (2.5)	16 (7.6)	0.17
Hyperlipidemia	11 (3.8)	8 (10)	3 (1.4)	0.002
Epilepsy	4 (1.4)	3 (3.8)	1 (0.5)	0.07

Table 2 presents a comparison between cases and controls. Patients with narcolepsy had a higher prevalence of hypothyroidism and hyperlipidemia and a higher prevalence of high-risk for OSA than controls.

Table 3 presents comorbidities that had a significant association with both NT-1 and NT-2. As patients with NT-1 were younger and had a higher BMI than controls, we adjusted for age and BMI (Table 2). Hypothyroidism and high risk of OSA were more common among patients with NT-1 than controls in the crude (model-0) and the adjusted (model-1) analysis. In model-1 for NT-1, the OR for hypothyroidism was 5.49 (95% CI, [0.8 - 38.6]), and the OR for high risk for OSA was 69.99 ((95%CI [20.6 -237.4]). Among patients with NT-2, after adjustments (model-1), hypothyroidism and high risk of OSA were also more common among cases than controls (OR 12.5, 95%CI [1.6-97.7], and OR 33.3, 95%CI [8.2-135.7], respectively).

**Table 3.** A comparison between the demographic characteristics and comorbid conditions in narcolepsy (NT-1, NT-2), and controls

Mean ± SE/n (%)				
Variable	NT1(n=56)	NT2 (n=24)	Control (n=222)	P-Value
Sex (Male)	46 (82.1)	21 (87.5)	158 (71.2)	0.076
Age (years)	32 ± 1.9	40.6 ± 2.4	36.6 ± 0.9	0.001 *
BMI (kg/m^2^)	29.7 ± 0.9	25.8 ± 0.9	27.7 ± 0.4	0.022 *
Comorbidities				
Hypothyroidism	3 (5.4)	2 (8.3)	2(0.9)	0.049 *
OSA	29 (51.8)	9 (37.5)	4 (1.8)	<0.001 *
**Model 0**				
**Variable**	**NT1 vs Control**	**NT2 vs Control**		
	**OR** **[95% C.I.]**	**P-Value**	**OR** **[95% C.I.]**	**P-Value**
Sex (Male)	1.9 [0.9 - 3.9]	0.1	2.8 [0.8 - 9.8]	0.1
Age (years)	0.97 [0.9 - 0.99]	0.02 *	1.02 [0.99 - 1.05]	0.2
BMI (kg/m^2^)	1.1 [1.003 - 1.1]	0.04 *	0.92 [0.8 - 1.01]	0.1
Comorbidities				
Hypothyroidism	6.2 [1.02 – 38.2]	0.048 *	10 [1.3 – 74.5]	0.025 *
OSA	58.5 [19.1 -179.3]	<0.001 *	32.7 [9.01 –118.65]	<0.001 *
**Model 1**				
**Variable**	**NT1 vs Control**	**NT2 vs Control**		
	**OR** **[95% C.I.]**	**P-Value**	**OR** **[95% C.I.]**	**P-Value**
Sex (Male)				
Age (years)				
BMI (kg/m^2^)				
Comorbidities				
Hypothyroidism	5.49 [0.8 - 38.6]	0.087	12.5 [1.6 – 97.7]	0.016 *
OSA	69.99 [20.6 -237.4]	<0.001 *	33.3 [8.2 – 135.7]	<0.001 *

**Model 0: crude associations. Model 1: adjusted for sex, age, and BMI.**

* The difference between NT-1 and controls is significant.

## DISCUSSION

This is the first study to assess medical comorbidities in Arab (Saudi) patients with narcolepsy. Hypothyroidism, high-risk for OSA, and hyperlipidemia were more common in narcolepsy patients than controls. However, after dividing narcolepsy patients into NT-1 and NT-2 and adjusting for age and BMI for NT-1 patients, hypothyroidism and high risk of OSA were significantly associated with narcolepsy.

Previous studies have suggested an association between orexin and thyroid-stimulating hormone (TSH); however, conflicting data have been reported^13^. It has been postulated that orexin may have a role in the control of the hypothalamic-pituitary-adrenal (HPA)^13^. A previous case-control study assessed the effects of orexin on the circulating TSH levels in seven patients with narcolepsy and reported lower circulating TSH levels in orexin-deficient patients with narcolepsy^13^. The thyroid releasing hormone (TRH) is synthesized in the paraventricular nucleus (PVN); however, orexinergic neurons widely project to the PVN^14^. Therefore, it is possible that orexinergic-neurons influence the TRH neuronal activity; nevertheless, the nature of this interaction is not known, whether excitatory or inhibitory. Cohen et al. (2018)^5^, in a community sample of narcolepsy, reported a strong association between narcolepsy and thyroid disease (OR 3.07; 95%CI: 1.19-7.90). The current and previously published data suggest an association between narcolepsy and thyroid disease, which is probably related to a co-existing or causal autoimmune disorder^15^.

However, in this study, a higher risk of hypothyroidism was present in NT-2 than NT-1 (OR: 12.5 vs. 6.33). It would be expected that hypothyroidism (an autoimmune disease) would have a higher prevalence in patients with NT-1. However, a higher prevalence of autoimmune diseases in NT-2 is supported by two recent studies that assessed the association between narcolepsy and autoimmune diseases. Alomar et al. (2019)^1^ and Barateau et al. (2017)^16^, compared the frequencies of autoimmune diseases in narcolepsy patients (NT-1 and NT-2) and healthy controls. Both studies revealed that NT-1 was not associated with an increased risk of autoimmune disorders^1,16^. On the other hand, both studies reported a higher frequency of autoimmune diseases in NT-2 patients compared to healthy controls. It is possible that NT-1 has distinctive autoimmune pathophysiology that is not associated with an increased risk of other autoimmune diseases^16^. The findings of the current study and the two previously published studies suggest a susceptibility to activate the immune system in patients with NT-2^16,17^. Therefore, future investigations are needed to explore the associations between NT-2 and autoimmune diseases.

The current results concur with previous studies demonstrating an association between narcolepsy and OSA^18^. An older study in Saudi Arabia reported that 25% of narcolepsy patients had OSA confirmed by PSG^19^. A Danish study collected data from a national information database three years before and after a narcolepsy diagnosis^20^. Before and after the narcolepsy diagnosis, the risk of OSA was (OR: 44.5, 95%CI: 13.1-151.3, and OR: 19.2, 95%CI: 7.7-48.3, respectively)^20^. Similar results were reported by a large American retrospective study of the Truven Health Analytics MarketScan Research Databases, which reported a high risk of comorbid OSA in narcolepsy patients (OR: 18.7, 95%CI: 17.5-20.0)^4^. Another American community-based study that included age- and sex-marched controls reported the risk of OSA in narcolepsy patients at diagnosis to be (OR: 69.25, 95%CI: 9.3-517.99)^5^. In general, narcolepsy is associated with increased BMI, which may increase the risk of OSA. However, in this study, we selected a control group matched for BMI^21^. Even after adjusting for BMI in NT-1 patients, OSA had a strong association with narcolepsy.

Additionally, this study revealed that OSA was more associated with NT-1 than NT-2. OSA is known to be associated with increased body weight as indicated above; therefore, it is possible that NT-1 patients had a higher association with OSA due to a significantly higher BMI than NT-2 patients. This could be one of the reasons for the increased association between NT-1 and OSA. A previous study that assessed the association between narcolepsy with OSA demonstrated that narcolepsy patients with comorbid OSA were heavier than those without OSA^22^. Nevertheless, a recent study reported a higher prevalence of REM-related OSA among NT-1 patients than NT-2 patients, despite comparable BMI in the two groups, suggesting other possible mechanisms for the association between NT-1 and OSA^23^. Several clinical studies reported a link between plasma orexin-A levels and OSA. An earlier study reported that plasma orexin-A levels were correlated negatively with the AHI^24^. Moreover, lower plasma orexin-A levels improved with continuous positive airway pressure in patients with severe OSA^25^. It has been hypothesized orexin level may affect pharyngeal cavity patency; however, this hypothesis needs to be tested^26^. Future research should focus on the role of orexin in upper airway control and patency.

As OSA may present with daytime sleepiness, and as OSA is more common than narcolepsy, the diagnosis of narcolepsy may be overlooked in some patients with OSA, resulting in a delayed diagnosis^27^. Therefore, the possibility of narcolepsy in patients with excessive daytime sleepiness should always be considered in the differential diagnosis, even in the absence of the classic tetrad of narcolepsy. Narcolepsy patients with comorbid OSA should receive treatment for both disorders. Although sleepiness may improve with positive airway pressure therapy in patients with narcolepsy and comorbid OSA^22^, narcolepsy-related sleepiness will not resolve with positive airway pressure therapy. Therefore, sleepiness in narcolepsy patients should be treated by pharmacotherapy, and positive airway pressure therapy should remain an adjunctive therapy to resolve the respiratory events.

Hyperlipidemia was also more common among patients with narcolepsy in this study than controls, where 10% of the patients, despite their relatively young age, were diagnosed with hyperlipidemia. Cohen et al. (2018)^5^ has reported similar results with increased risk of hyperlipidemia in patients with narcolepsy (OR: 2.49, 95%CI: 1.05-5.92). Ohayon (2013)^8^ reported a prevalence of hyperlipidemia of 11.6% among patients with narcolepsy, which is comparable to our results (10%). It is not known why there is an association between narcolepsy and hyperlipidemia. One possible mechanism is the increased prevalence of thyroid diseases among narcolepsy patients. Future research should explore the mechanisms linking narcolepsy and hyperlipidemia.

This brief report has strengths and limitations. Strengths include the fact that the study was conducted in one of the major referral centers for narcolepsy in the country. Furthermore, no study has assessed comorbid medical disorders in Arab patients with narcolepsy. Limitations include the fact that medical disorders were self-reported, although we also checked medical records (when feasible) to confirm the comorbid diagnoses. Nevertheless, the wmethod used in the current study has been utilized in previous studies that assessed comorbid conditions in narcolepsy^16,28,29^. Another limitation is the fact that we did not measure cerebrospinal fluid orexin levels in NT-2 patients. Finally, the data are reported from a single-center, and the sample size is relatively small to have adequate power to detect differences between groups. Therefore, a multicenter study involving several Arab countries is needed to confirm the current findings.

## CONCLUSION

In summary, Arab (Saudi) narcolepsy patients had a higher association with hypothyroidism, hyperlipidemia, and a higher risk of OSA. These findings concur with data from Western societies. However, prospective multicenter larger studies should be conducted to verify the findings of this brief report.
